# Supplementary material for: BLTP3A is associated with membranes of the late endocytic pathway and is an effector of CASM
Source: bioRxiv. 2025 Apr 3:2024.09.28.615015. Originally published 2024 Sep 28. Preprint. [Version 3] doi: 10.1101/2024.09.28.615015 (PMC11463362; doi:10.1101/2024.09.28.615015)

bioRxiv preprint doi: https://doi.org/10.1101/2024.09.28.615015; this version posted April 3, 2025. The copyright holder for this preprint (which was not certified by peer review) is the author/funder, who has granted bioRxiv a license to display the preprint in perpetuity. It is made available under aCC-BY 4.0 International license.

SUPPLEMENTAL FIGURE LEGENDS

Supplemental Figure 1

bioRxiv preprint doi: https://doi.org/10.1101/2024.09.28.615015; this version posted April 3, 2025. The copyright holder for this preprint (which was not certified by peer review) is the author/funder, who has granted bioRxiv a license to display the preprint in perpetuity. It is made available under aCC-BY 4.0 International license.

(A) Live fluorescence images (inverted grays) of RPE-1 cells expressing either GFP-Rab45 (left), BLTP3A-mRFP (center), or both proteins together (only BLTP3A is shown) (right) as indicated. Scale bar, 5 μm. High-magnification scale bar, 2 μm.
(B) Genomic sequence of the edited BLTP3A locus (insertion of the V5 epitope) in A549 cell. Blue, small Gly-Ser linkers; green, V5 epitope sequence.
(C) AlphaFold prediction of BLTP3A. The site where the V5 epitope (V904) was inserted is indicated. The long disordered sequence and the C-terminal helix are shown in gray.
(D) Left: Fluorescence image of an RPE-1 cell expressing exogenous BLTP3B-mRFP (inverted grays) and immunolabeled with antibodies against endogenous VAMP7 (shown at right in the high magnification of the squared region in the main field). Scale bar, 5 μm. Right: zooms of different RPE-1 cells expressing exogenous BLTP3B-mRFP (magenta) and immunolabeled with antibodies (green) against endogenous VAMP4 or ATG9A. Individual channels are shown as inverted grays. Merge of channels on bottom. Scale bar, 1 μm.
(E) Fluorescence images of RPE-1 cells expressing the indicated BLTP3A-mRFP construct. Scale bar, 5 μm.
(F) Live fluorescence images (inverted grays) of RPE-1 cells expressing exogenous GFP-LRRK1^{K746G} (left) and BLTP3A-mRFP (right). Scale bar, 10 μm.
(G) Western blot of lysate of RPE-1 cells expressing exogenous RFP-LRRK1^{K746G} or RFP-LRRK1^{D1409A} for RFP (to detect LRRK1 fusions), Rab7, phospho-Rab7 S72, and alpha-tubulin as a loading control.

Supplemental Figure 2

(A) AlphaFold3 multimer prediction of full-length MAP1LC3B (green) and a.a. 1110-1150 of BLTP3A (magenta). Arrows indicate key residues of the LIR motif of BLTP3A.
(B) AlphaFold3 multimer predictions of mATG8 proteins and a.a. 1110-1150 of BLTP3A with and without the LIR motif (ΔLIR).
(C) Time-series of live fluorescence images (inverted grays) of BLTP3A-mRFP and GFP-LC3B before and after addition of GPN. Arrowheads point to lysosomes where BLTP3A and LC3B decorate the entire profile upon addition of GPN. Time, seconds. Scale bar, 5 μm.
(D) Genomic sequence of the edited BLTP3A locus in A549 cell. Blue, gRNA; green, PAM; red, indel mutations.
(E) Quantification of relative LAMP1 expression from western blots (n=3) of Figure 7A. Error bars indicate the standard error of the mean (SEM).

Supplemental Movie 1
FIB-SEM reconstruction of GFP-LC3B and BLTP3A-mRFP-positive lysosomes in an RPE-1 cell 15 min after LLOMe addition (from Figure 5E). Reconstructed organelle colors: lysosome, dark green; ER, yellow; mitochondria, blue; small vesicles, magenta; large vesicles, green. Scale bar, 1 μm.

Supplemental Movie 2
FIB-SEM image stack of the region used for the reconstruction in Figure 5E and Supplemental Movie 1. Lysosomes are pseudo-colored dark green. Scale bar, 1 μm.

bioRxiv preprint doi: https://doi.org/10.1101/2024.09.28.615015; this version posted April 3, 2025. The copyright holder for this preprint (which was not certified by peer review) is the author/funder, who has granted bioRxiv a license to display the preprint in perpetuity. It is made available under aCC-BY 4.0 International license.

Supplemental Figure 1

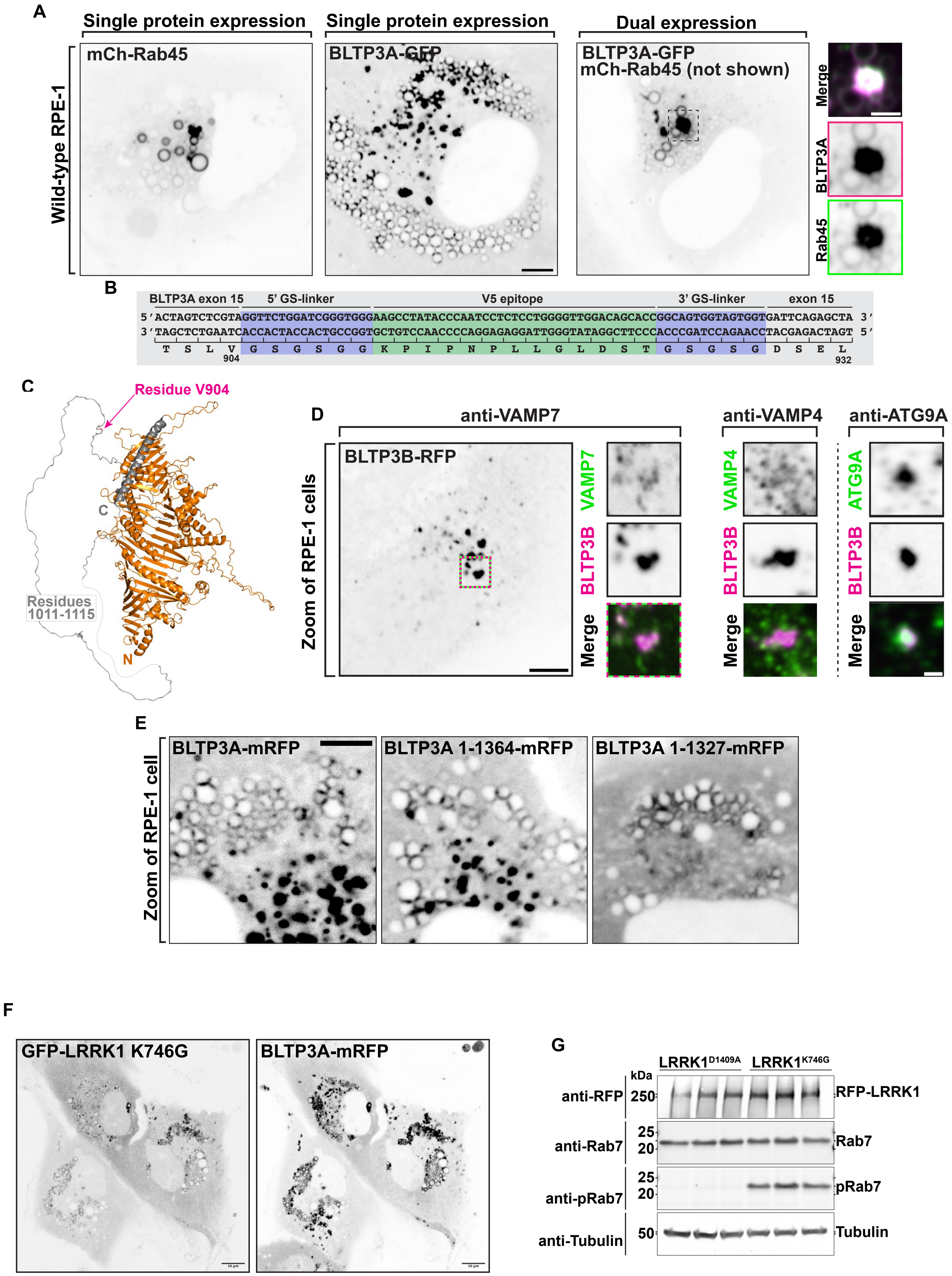

bioRxiv preprint doi: https://doi.org/10.1101/2024.09.28.615015; this version posted April 3, 2025. The copyright holder for this preprint (which was not certified by peer review) is the author/funder, who has granted bioRxiv a license to display the preprint in perpetuity. It is made available under aCC-BY 4.0 International license.

Supplemental Figure 2

A

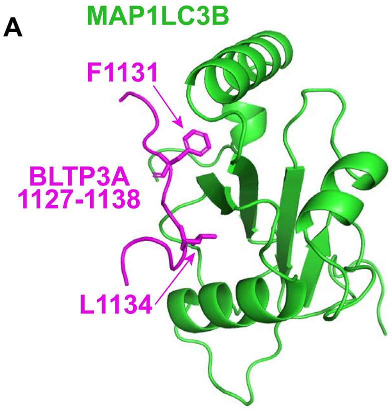

B

AF3 predictions of BLTP3A with mAtg8 proteins

BLTP3A:	1110-1150		1110-1150 ΔLIR	
	ipTM	pTM	ipTM	pTM
MAP1LC3A	0.66	0.7	0.31	0.69
MAP1LC3B	0.56	0.68	0.32	0.7
MAP1LC3C	0.57	0.62	0.32	0.62
GABARAP	0.74	0.76	0.55	0.74
GABARAPL1	0.73	0.75	0.52	0.72
GABARAPL2	0.72	0.74	0.53	0.73

C

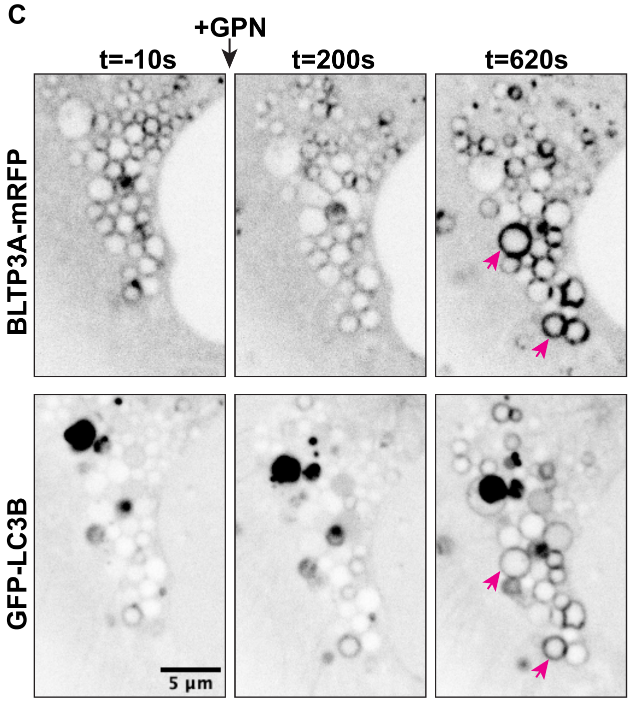

D

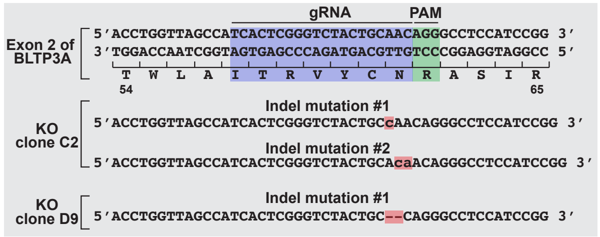

E

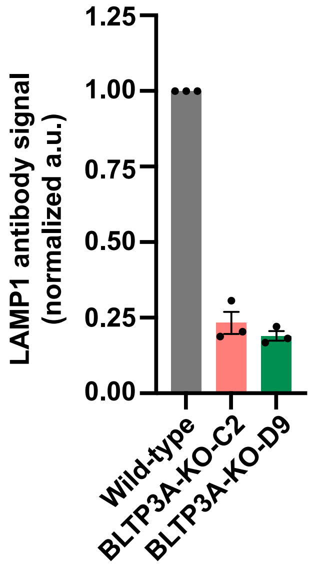

Supplement: Supplement 1 [file NIHPP2024.09.28.615015v3-supplement-1.pdf]
